# 

**Published:** 1914-10-10

**Authors:** 


### Buildings Contemplated.

Brandon.—The Brandon and Byshottles Urban District Council have agreed to purchase a site for an isolation hospital.

Holsworthy.—The Holsworthy Urban District Council have appointed a committee to confer with the Rural District Council, with a view to providing a joint isolation hospital.

Ipswich.—An inquiry has been held by the Local Government Board into an application of the Corporation for permission to borrow £8,000 for the extension of the isolation hospital.

Rugby.—The Guardians have ordered the preparation of plans for alterations to the workhouse.

Walker.—The Newcastle-on-Tyne Corporation have approved a contract at £9,200 for a new tuberculosis hospital near Walker Hospital.

Rates of Subscription : Twelve months, 6/6 ; Six months, 4/- ; Three months, 2/-, post free to any address in the United Kingdom. Abroad, Twelve months, 8/8; Six months, 5/-; Three months, 2/6, post free.—Address The Subscription Dept., "The Hospital," The Hospital Building, 28/29 Southampton Street, Strand, London W.C.

